# Temporomandibular Joint Disorders Associated With Psoriatic Arthritis: A Report of Three Cases

**DOI:** 10.7759/cureus.32959

**Published:** 2022-12-26

**Authors:** Peeyush Shivhare, Aayushma Chapagain Ghimire, Karishma ., Abhishek Singh, Vyakhya Gupta

**Affiliations:** 1 Oral Medicine and Radiology, All India Institute of Medical Sciences, Patna, Patna, IND; 2 Dentistry, Nobel Medical College Teaching Hospital, Biratnagar, NPL; 3 Oral and Maxillofacial Surgery, Shaikh-Ul-Hind Maulana Mahmood Hasan Medical College, Saharanpur, IND; 4 Oral and Maxillofacial Surgery, All India Institute of Medical Sciences, Patna, Patna, IND

**Keywords:** temporomandibular joint, temporomandibular joint disorders, psoriatic arthritis, psoriasis, caspar criteria

## Abstract

Psoriatic arthritis is chronic inflammatory arthritis (seronegative) associated with psoriasis. Patients may present with different clinical symptoms as it progresses and involves multiple joints one after the other in an erratic pattern of aggravation and remission. A thorough examination is necessary for an accurate diagnosis and appropriate therapy of the disorder since it exhibits similar characteristics as reactive arthritis, rheumatoid arthritis, and ankylosing spondylitis. There haven't been many instances of the temporomandibular joint being involved, but records have to be maintained properly to assess its role in psoriatic arthritis. Here, we present three cases of temporomandibular joint disorders associated with psoriatic arthritis.

## Introduction

The temporomandibular joint also termed the ginglymoarthrodial joint consists of the glenoid fossa of the temporal bone and head of the mandibular condyle, articular disc, and associated muscles and ligaments [1]. As per the Diagnostic Criteria for Temporomandibular Disorders (DC/TMD), temporomandibular disorders include joint pain (arthralgia, arthritis), joint disorders (disc disorders, hypermobility disorders), joint diseases (degenerative joint diseases, systemic arthritides, neoplasm), fractures, and congenital disorders [2]. The term arthritis denotes any inflammatory condition of the joint, varying from osteoarthritis to systemic arthritides [3]. Systemic or inflammatory arthritides include rheumatoid arthritis (RA), systemic lupus erythematosus (SLE), gout, pseudo-gout, ankylosing spondylitis (AS), and psoriatic arthritis (PsA) [4].

Psoriasis is a systemic, immune-mediated, chronic inflammatory skin disease that affects the skin, nails, scalp, and mucous membranes. It affects 1-3% of the world’s population [5]. Geographic tongue, fissured tongue, periodontitis, candidiasis, and temporomandibular joint disorders (TMDs) are the most common oral manifestation [6].

Psoriatic arthritis is a chronic inflammatory arthritis (seronegative) associated with psoriasis. It commonly involves the distal interphalangeal joints, joints of knees, elbows, shoulders, ankles, spine, toes, and rarely TMJ [7,8]. Psoriatic arthritis (PA) seems to be associated with 5-24% of psoriatic patients. They develop at the same time after or even prior to skin findings [7].

Different genetic and immunologic studies confirm different immune mediators, potentially involved in the pathogenesis of psoriatic arthritis such as interleukin-1 (IL-1), IL-1b, IL-6, IL-9, IL-12, IL-15, IL-17A, IL-17F, IL-18, IL-19, IL-20, IL-21 IL-22, IL-23, IL-26, IL-34, IL-38, tumor necrosis factor (TNF), interferon-gamma (IFN-γ), granulocyte-macrophage colony-stimulating factor (GM-CSF), reactive oxygen species (ROS), nitric oxide, matrix metalloproteinases (MMPs), orthopantomogram (OPG), receptor activator of nuclear factor kappa-Β ligand (RANKL), cathepsin K, a disintegrin and metalloproteinase with thrombospondin motifs (ADAMTS), and nitric oxide. These mediators are released by different immunological (mast cells, dendritic cells, macrophages, mucosal-associated invariant T cell, Th 1, Th9, Th 17/17 type cells, Th 22), and nonimmunological cells (fibroblast-like synoviocytes, osteoblasts, osteoclasts, chondrocytes) [5]. Out of these, all IL-23-IL-17 axis and type 17 cells are central to the etiopathogenesis of PsA [5,6].

An observational study performed by Crincoli V et al. (2015) concluded that psoriasis seems to play a role in TMJ disorders, causing an increase in orofacial pain and an altered chewing function [9]. Here, we present three different scenarios of TMD encountered in psoriatic arthritis patients.

## Case presentation

Case 1

**Figure 1:**
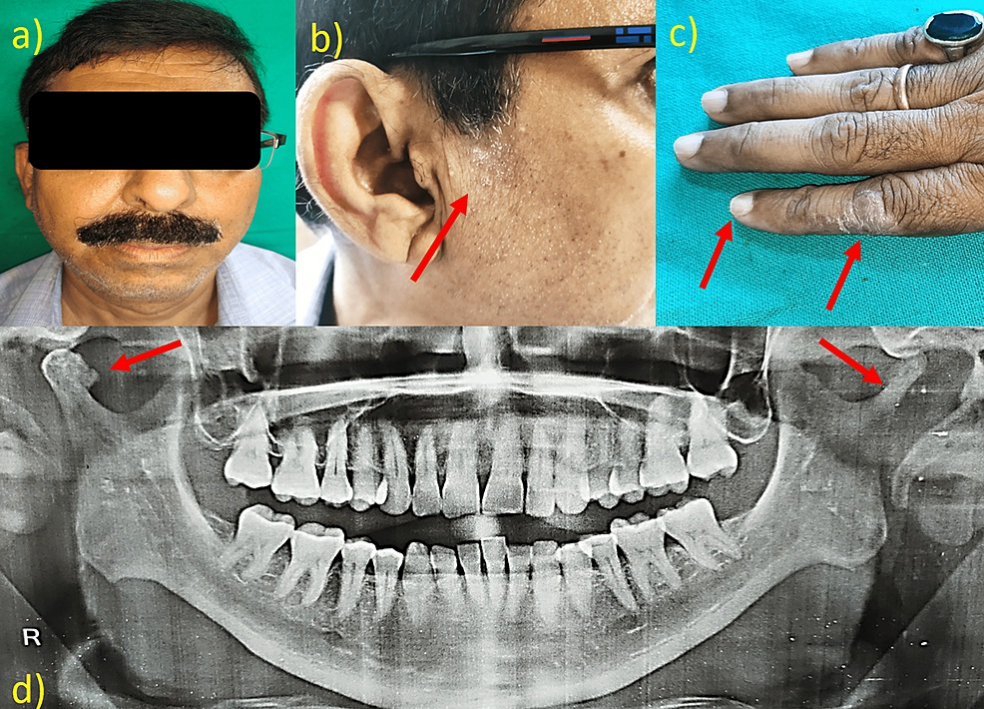
a) No significant asymmetry, b) presence of psoriatic patch over the facial region, c) involvement of nails and fingers, d) the panoramic radiograph reveals bony erosion with osteophyte in relation to the right condyle while a sharpened pencil appearance in relation to the left TMJ TMJ: temporomandibular joint

A 48-year-old male patient who had been complaining of temporomandibular joint (TMJ) pain for a month visited the Department of Dentistry, AIIMS, Patna. The pain was progressive, dull hurting, measuring moderately on the visual analog scale (VAS - 6), non-radiating, aggravated by chewing, and relieved with medications. There was no history of prior traumatic events, fever, or orofacial infections. According to medical records, the patient had psoriasis for five years and was receiving therapy for it (methotrexate). No obvious facial asymmetries were found during the extraoral assessment (Figure 1a). Psoriatic patchy rashes were seen on the forehead, behind the ears, and above the hairline (Figure 1b). The nail of both hands nails exhibits isolated pitting (Figure 1c). TMJ discomfort with a left-side deviation and a clicking sound was noted throughout the evaluation. Based on the clinical symptoms, a provisional diagnosis of psoriatic arthritis along with differential diagnoses of osteoarthritis and rheumatoid arthritis were provided. Osteophyte and bony degradation of the right TMJ were observed in the panoramic radiograph (Figure 1d). In the left TMJ, bone erosion was more pronounced and was beginning to resemble a sharpened pencil. When blood investigations were performed, the erythrocyte sedimentation rate (ESR) was increased but the rheumatoid factor test was negative. The final diagnosis of psoriatic arthritis was made per the clinical, radiographic, and CASPAR (classification criteria for psoriatic arthritis) criteria. Since the patient was already receiving immunomodulators (methotrexate), no more pharmaceutical care was anticipated, and the case was forwarded to a rheumatologist. Prednisolone 10 mg once daily was prescribed together with methotrexate 15 mg once a week, ranitidine 150 mg twice daily, and calcium with vitamin D3 initially for two months to the patient at the rheumatic clinic. Additionally, a soft diet and a little mouth opening were prescribed to the patient. After a month, the patient symptomatically improved during the follow-up visit (VAS - 02). The patient was instructed to keep taking her medicine and see her rheumatologist for regular checkups.

Case 2

**Figure 2:**
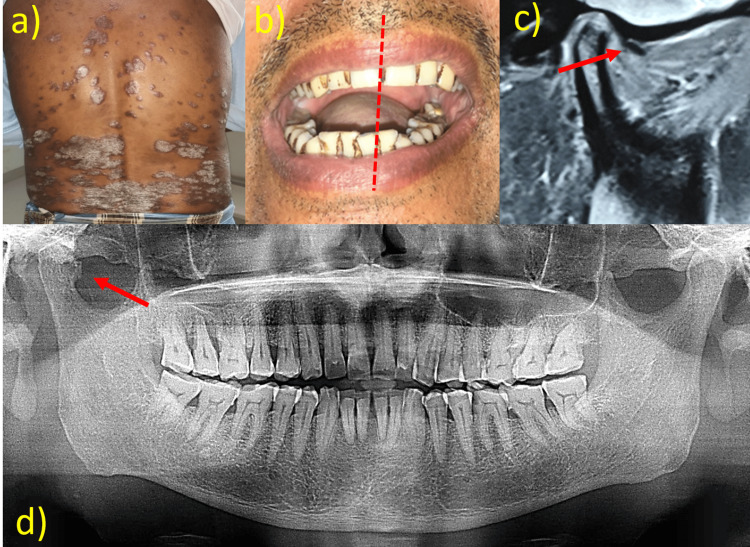
a) Presence of psoriatic patch over the back of the body, b) restricted mouth opening along with deflection on the right side, c) MRI of the right TMJ reveals an anteriorly placed articular disc, d) panoramic radiograph reveals an osteophyte with bony erosion and Ely’s cyst in relation to the right condyle TMJ: temporomandibular joint

For the past two weeks, a 54-year-old male patient had been complaining of restricted mouth opening and pain while wide mouth opening. initially, there was a history of joint noise with mild pain. Gradually, mouth opening got limited with painful mouth opening. The patient also describes past joint discomfort that was worst upon rising in the morning and when eating. According to the patient's medical history, psoriatic plaque is present on the skin, hands, nails, and scalp, and has been under the care of a rheumatologist for the past ten years. Non-steroidal anti-inflammatory drugs (NSAIDs) and methotrexate were being administered to the patient. Psoriatic patchy rashes were seen all over the back, neck, legs, and hands, along with nail dystrophy (Figure 2a). There was no facial asymmetry found during the extraoral examination. Mouth opening was limited to 20 mm with a rightward deflection (Figure 2b). The patient experienced discomfort during jaw opening, protrusion, and lateral movement during clinical functional manipulation. The VAS score was 5 in the relaxed posture and 7 when the patient was being palpated. The end-feel movement test resulted in a negative/hard-end feel since there was no discernible increase in the forced opening. A preliminary diagnosis of anterior disc displacement without reduction was made based on the clinical characteristic. Panoramic radiographs and MRI were recommended. An anteriorly displaced articular disc of the right TMJ was shown on MRI (Figure 2c). The panoramic radiograph revealed condyle erosion along with osteophytes and Ely’s cyst on the right TMJ (Figure 2d). Anterior disc derangement without reduction owing to psoriatic arthritis was diagnosed based on clinical and radiographic symptoms. The patient was recommended to keep taking the psoriasis medication, and arthrocentesis was planned for the patient's TMJ problem. The mouth opening was increased to 35 mm after arthrocentesis. A different patient was sent to the rheumatology department for further assessment of the dermatologic lesion. The patient improved symptomatically over the follow-up period.

Case 3

**Figure 3:**
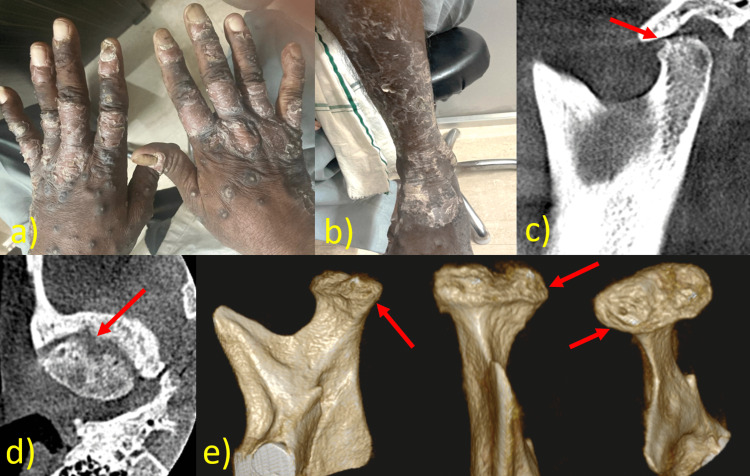
a) Presence of psoriatic patch over hands and feet, b) oblique sagittal section shows concave-shaped remodeling with multiple osteophytes of the superior surface, c) oblique axial section shows an osteophyte in the superior and anterior surfaces along with erosion. d) 3D CBCT CBCT: cone beam computed tomography

A 43-year-old female patient reported a complaint of pain on the right side of her face while biting on hard objects and on mouth wide opening for the past two weeks. The pain was progressive and of modest intensity (VAS - 06). There was no evidence of prior trauma. On the medical history form, the patient also mentions a history of psoriasis for which they had been taking medication for four years (methotrexate). Upon skin inspection, psoriatic plaque and nail degeneration were discovered (Figures 3a, 3b). On extraoral inspection, the patient's face was symmetrical with a slight right-sided deviation of the mouth when it was opened wide. The DC/TMD criteria were followed during the clinical evaluation. Up to 25 mm, the patient was able to expand their mouth without any tenderness, further mouth opening was painful but mouth opening was possible with a little assistance (soft end feel was positive). On palpation, the right superficial and deep masseter along with the lateral pterygoid were painful (VAS - 09) and had tender points. Mild tenderness (VAS - 03) was seen in relation to the right and left TMJs during TMJ palpation. The panoramic radiograph revealed mild condylar erosions and prominent osteophytes of the right TMJ. Cone beam computed tomography (CBCT) revealed concave remodeling/erosion of the superior surface with multiple small osteophytes (Figures 3c-3e). Following a blood test, the complete blood picture (CBP) revealed a normal ESR of 40 mm/hr and a negative Rh factor. Based on clinical (CASPAR criteria) and radiological features, a diagnosis of myofascial pain dysfunction syndrome related to psoriatic arthritis was given. A muscle relaxant (chlorzoxazone coupled with piroxicam 20 mg) along with transcutaneous electrical nerve stimulation (TENS) therapy was recommended to the patient. In addition, the patient was forwarded to the rheumatologist's office for additional assessment of the dermatologic lesion. During the follow-up period, the patient's symptoms improved.

## Discussion

As per the DC/TMD recommendation, arthritis related to a systemic disease and categorized as “systemic arthritides” includes rheumatoid arthritis (RA), systemic lupus erythematosus (SLE), gout, pseudo-gout, ankylosing spondylitis (AS), and psoriatic arthritis (PsA) [2,4].

Psoriasis and joint disorders are two capabilities of the persistent musculoskeletal circumstance referred to as PsA [7,8]. Various criteria have been published previously for differentiating psoriasis arthritis from other similar clinical entities such as Moll & Wright (M & W), Bennett's, Vasey and Espinoza (V & E), Fournié's, European Spondyloarthropathy Study Group (ESSG), McGonagle, Gladman, and most recently, the CASPAR Study Group [10,11].

A study concluded the high sensitivity and high specificity (91.4% and 98.7%, respectively) of the CASPAR criteria for the classification of PsA, thus a patient needs to follow the CASPAR criteria for categorization to “psoriatic arthritis” [11]. The CASPAR criteria are summarized in Table 1 [10]. All cases of our study followed the CASPER criteria.

**Table 1:** CASPAR criteria Inflammatory articular disease with three or more of these points is considered to be psoriatic arthritis.

Category	Points
Current psoriasis	Score 2
Personal history of psoriasis	Score 1
Family history of psoriasis: psoriasis in a first- or second-degree relative	Score1
Psoriatic nail dystrophy	Score 1
RF negative	Score 1
Current dactylitis or History of dactylitis	Score 1
Radiological evidence of juxta-articular new bone formation	Score 1

Psoriatic arthritis follows a very similar pattern to rheumatoid arthritis in terms of joint erosion and impaired quality of life, and thus needs special attention to differentiate both. The differentiating features are summarized in Table 2 [12,13].

**Table 2:** The differentiating features between psoriatic arthritis and rheumatoid arthritis CCP: cyclic citrullinated peptide, HLA: human leukocyte antigen, IL: interleukin, TNF-α: tumor necrosis factor-alpha, CD: cluster of differentiation

	Psoriatic arthritis	Rheumatoid arthritis
Joint involvement	Mostly asymmetrical	Mostly symmetrical
Typically affected joints	distal interphalangeal joints along with other joints too	Distal interphalangeal joints
Number of affected joints	Oligoarthritis	Polyarthritis
Enthesitis	In 60-80% of cases	Not typical
Dactylitis	In 30% of cases	Not typical
Involvement of the spine	Axial spine	Cervical spine
Skin/nails	In 80% of cases, plaque vulgaris, pustular psoriasis; in 60 % of cases, onycholysis, psoriatic onychodystrophy	Not typical
Serology	CCP and rheumatoid factor are negative	CCP and rheumatoid factor are positive
Radiological findings	More bone proliferation (osteophytes) compared to erosion. (Osteophytes component was appreciated in our 3 cases)	More bone erosion with punched out region compared to proliferation.
Most involved genetic allele	HLA-B27 alleles	HLA-DRB1 alleles
Key cytokines	IL-17, IL-23, IL-22, IL-1β, IL-6, interferon-γ and TNF-α	TNF-α, IL-6, IL-1, IL-22, IL-33, chemokine ligand 11 and chemokine (C-X-C motif) ligand
Levels of IL-17 and CD8+ T cells	Elevated	Not elevated

The characteristics clinical features of psoriatic arthritis involving TMJ are occlusal derangement, pain, pre-auricular swelling, deviation of the jaw while opening/closing, restricted mouth opening, masticatory muscle pain, and inability to adequately open or close the mouth [7,8]. While radiographic manifestations include erosion of the condyle, presence of a proliferative component (osteophyte), narrowing of the joint space, the anterior position of the condylar head, erosion of the head and roof of the glenoid fossa, and flattening of the articular eminence [14].

In our case series, the first case was psoriatic arthritis, the second case had anterior disc derangement secondary to psoriatic arthritis, and the last one was diagnosed with myofascial pain dysfunction syndrome secondary to psoriatic arthritis. Other than routine treatment of psoriases such as antimalarials, gold, methotrexate, sulfasalazine, azathioprine, and cyclosporin A. The TNF inhibitor, etanercept, and TMJ involvement need special care and attention [14,15]. The treatment should be incorporated to eliminate the secondary effects of the arthritic change such as anterior disc displacement and muscle pain. The primary and most essential step in treating TMJ issues is pain. Conservative remedy includes muscle relaxants, non-steroidal drugs (NSAIDs), physical therapy such as TENS, occlusal splints, low-level laser therapy, low-level ultrasound therapy, trigger point injections, and arthrocentesis [15,16].

## Conclusions

Psoriasis patients may present with different clinical symptoms as it progresses and involves multiple joints one after the other in an erratic pattern of aggravation and remission. Psoriatic arthritis is a chronic inflammatory arthritis (seronegative) associated with psoriasis. A thorough examination is necessary for an accurate diagnosis and appropriate therapy of the disorder. Psoriatic arthritis follows a very similar pattern to rheumatoid arthritis in terms of joint erosion and impaired quality of life, and thus it needs special attention to differentiate both.

## References

[1] Shivhare P, Singh V, Giri R, Singh A, Penumatcha MR, Taparia N, Sabah N (2019). Prevalence of temporomandibular disorders among Nepalese population. J Chitwan Med Coll.

[2] Schiffman E, Ohrbach R, Truelove E (2014). Diagnostic Criteria for Temporomandibular Disorders (DC/TMD) for Clinical and Research Applications: recommendations of the International RDC/TMD Consortium Network* and Orofacial Pain Special Interest Group†. J Oral Facial Pain Headache.

[3] Shivhare P, Shankarnarayan L, Singh A, Haidry N (2015). Temporomandibular Joint manifestations in rheumatoid arthritis: a case report. IJSS Case Reports Rev.

[4] Poudel P, Goyal A, Lappin SL (2022). Inflammatory Arthritis. https://pubmed.ncbi.nlm.nih.gov/29939526/.

[5] Stober C (2021). Pathogenesis of psoriatic arthritis. Best Pract Res Clin Rheumatol.

[6] Costa AA, Cota LO, Mendes VS, Oliveira AM, Cyrino RM, Costa FO (2021). Impact of oral lesions on the quality of life of psoriatic individuals: a case-control study. Oral Dis.

[7] Okkesim A, Adisen MZ, Misirlioglu M (2017). Temporomandibular joint involvement in psoriatic arthritis. Niger J Clin Pract.

[8] R R, Malarkodi T, Azariah E, Warrier AS (2021). The involvement of temporomandibular joint in psoriatic arthritis: a report of a rare case. Cureus.

[9] Crincoli V, Di Comite M, Di Bisceglie MB, Fatone L, Favia G (2015). Temporomandibular disorders in psoriasis patients with and without psoriatic arthritis: an observational study. Int J Med Sci.

[10] Congi L, Roussou E (2010). Clinical application of the CASPAR criteria for psoriatic arthritis compared to other existing criteria. Clin Exp Rheumatol.

[11] Taylor W, Gladman D, Helliwell P, Marchesoni A, Mease P, Mielants H (2006). Classification criteria for psoriatic arthritis: development of new criteria from a large international study. Arthritis Rheum.

[12] Merola JF, Espinoza LR, Fleischmann R (2018). Distinguishing rheumatoid arthritis from psoriatic arthritis. RMD Open.

[13] Behr M, Fanghänel J, Miehe B, Schreml S (2022). Psoriatic arthritis and the temporomandibular joint. Dtsch Zahnärztl Z.

[14] Kumar BV, Kumar AV, Shankar U (2011). Psoriatic arthritis of TMJ presenting as a first articular complaint in psoriasis. J Indian Acad Oral Med Radiol.

[15] Shivhare P, Shankarnarayan L, Singh A, Patil ST, Yadav M (2015). Role of immunomodulators in oral diseases. Int J Oral Health Med Res.

[16] Shivhare P, Parihar A Temporomandibular disorder. Textbook of Oral Medicine and Radiology. 2nd Edition.

